# Use of Optical Oxygen Sensors in Non-Destructively Determining the Levels of Oxygen Present in Combined Vacuum and Modified Atmosphere Packaged Pre-Cooked Convenience-Style Foods and the Use of Ethanol Emitters to Extend Product Shelf-Life

**DOI:** 10.3390/foods2040507

**Published:** 2013-11-18

**Authors:** Andreas W. Hempel, Dmitri B. Papkovsky, Joseph P. Kerry

**Affiliations:** 1 Food Packaging Group, School of Food and Nutritional Sciences, University College Cork, Cork, Ireland; E-Mail: a.hempel@umail.ucc.ie; 2 Department of Biochemistry, University College Cork, Cork, Ireland; E-Mail: d.papkovsky@ucc.ie

**Keywords:** modified atmosphere packaging, convenience foods, packaging, storage, sensory, oxygen sensors, ethanol emitter

## Abstract

O_2_ sensors were used to non-destructively monitor O_2_ levels in commercially packed pre-cooked, convenience modified atmosphere packaging (MAP) foods. A substantial level of O_2_ (>15%) was present in packs resulting in a shorter than expected shelf-life, where the primary spoilage mechanism was found to be mould. Various combinations of vacuum (0–0.6 MPa) and gas flush (0.02–0.03 MPa) (30% CO_2_/70% N_2_) settings were assessed as treatments that result in the desired shelf-life (28 days). This was achieved using the combined treatment of vacuum 0.35 MPa and gas flush 0.02 MPa which resulted in a reduction of 6%–9% O_2_ in all three samples (battered sausages (BS), bacon slices (BA), and meat and potato pies (PP)). Reduced O_2_ levels reflect the microbial quality of products, which has been successfully reduced. Duplicate samples of all product packs were produced using ethanol emitters (EE) to see if shelf-life could be further extended. Results showed a further improvement in shelf-life to 35 days. Sensory analysis showed that ethanol flavour and aroma was not perceived by panellists in two of the three products assessed. This study demonstrates how smart packaging technologies, both intelligent and active, can be used to assist in the modification of conventional packaging systems in order to enhance product quality and safety and through the extension of product shelf-life.

## 1. Introduction

Convenience foods can be defined as commercially prepared foods designed for ease of purchase, preparation and consumption [1]. Such food items can be single elements of a meal or can be complete food items. It is widely believed that the importance of convenience in food is still on the increase, at least in many countries, and that changing demographics are a major driver in this process [2,3]. Retailer focus and greater consumer demand for quality maintenance and shelf-life extension of retailed convenience-style food products continues to challenge the development of these food forms. The shelf-life of heat and serve or ready-to-eat foods is usually limited by two factors; microbial growth and the oxygen sensitivity of the product. Therefore, the two main requirements when packaging convenience-style products under modified atmosphere (MA) is that oxygen should be excluded and a fungistatic or bacteriostatic agent be present [4]. The uses of advanced technologies have been researched in an attempt to exert greater control over the production and stabilisation of convenience-style food products. Technologies such as radiation treatment [5] and high pressure processing [6,7] have been used to increase the shelf-life of convenience-style foods, but are associated with high costs and utilisation issues. Simple and more commonly used technologies such as packaging may prove to be cheaper and more practical in terms of exerting greater process control during the manufacture of convenience-style food products. Technologies like modified atmosphere packaging (MAP) and vacuum packaging are utilised across the food industry to create packaging solutions capable of providing a sufficient shelf-life for the chilled chain distribution of numerous food types. The use and manipulation of such packaging systems have not been exploited sufficiently and in combination with new packaging materials offer opportunities to enhance greater control around food production, especially in the area of convenience-style food production. The use of MAP involves the use of O_2_, CO_2_ and N_2_ in ratios that differ to normal atmospheric air. These altered concentrations retard deterioration processes and maintain foods in a “fresh” state for extended periods of time [8]. Reduced oxygen levels, to that found in air, is commonly applied to oxygen-sensitive food packaging in order to reduce or delay oxidation reactions in foods. Aerobic microbial growth and oxidation reactions are the principal mechanisms responsible for food deterioration. Elevated levels of CO_2_ are utilised for selective antimicrobial effects, primarily targeted towards food spoilage microorganisms [9]. Vacuum packaging is also utilised in extending shelf-life in foods applications, where the atmosphere that normally surrounds the food is removed. Vacuum packaging of food products can be seen as an effective means of eliminating possible biological and chemical contaminants from the space surrounding the food [10]. 

Smart packaging is generally defined as packaging that provides additional levels of useful functionality beyond protecting, containing and providing information about the product [8,11]. Smart packaging encompasses and incorporates intelligent and active packaging formats. A smart packaging component can be described as intelligent if it has the ability to sense the environment and communicate its findings with the buyer or consumer; for example an intelligent package is one that can monitor the safety and/or quality condition of a food product and provide early warning to the consumer or food manufacturer [12]. A form of intelligent packaging that has received much interest is that of optical oxygen sensors [13,14,15]. Fluorescent-based oxygen sensors represent the most promising systems to date for remote measurement of headspace gases in packaged products. A number of disposable oxygen sensing prototypes has been developed that can be produced at low costs and provide rapid determination of oxygen concentration [15,16]. Sensors normally consist of a fluorescent or phosphorescent dye encapsulated in a solid polymer matrix and added to a suitable support material. If present, molecular oxygen quenches the luminescent dye and can be quantified against predetermined calibrations. The process is reversible and yields no by-products [11]. Research utilising optical oxygen sensors across a wide range of foods has been extensively published. Foods ranging from MAP cheese [17], vacuum packed cheese [18], MAP and vacuum packed beef [19], cooked meats [20], MAP and vacuum packed chicken [21], as well as *sous vide* products [22] have been monitored for oxygen levels using non-destructive, reversible, optical oxygen sensors. Further research has been carried out in the bottled beverage sector, where oxygen levels were determined in pre-pasteurised beer [23]. The ability to non-destructively assess the levels of O_2_ present immediately after packaging can provide valuable information into the shelf-life and overall quality of the packaged food at any time point during the life-time of the product. Post packaging assessment could lead to the development of acceptable limits that could ensure product quality throughout shelf life and during storage periods [18]. 

Another form of smart packaging is that of active packaging. This is defined as packaging in which subsidiary constituents have been deliberately included in, or on, either the packaging material or the package headspace to enhance the performance of the package system [24]. Scavengers, emitters, absorbers and releasers are commonly utilised active packaging materials incorporated to food packaging applications to extend shelf-life. The use of ethanol is particularly effective against mould but can also inhibit the growth of yeasts and bacteria [25]. Ethanol sachets containing ethanol-release vapour imparts a preservative effect in the packaging headspace [26]. Many forms of ethanol emitting sachets have been patented and available for purchase including; Ethicap™, Antimould 102™ and Negamold™ (Freund Industrial Co. Ltd.,Tokyo, Japan) and Ageless™ type SE (Mitsubishi Gas Chemical Co. Ltd., Tokyo, Japan). However, limited applications of such technologies have been reported in the scientific literature.

The objective of this study was to assess various packaging technologies and assess their capacity to extend the shelf-life of a range of commercial convenience-style products that were found to have a reduced shelf-life primarily influenced by mould spoilage. The integration of oxygen sensors in food packs to monitor the levels of oxygen remaining in packs post packaging and the application of ethanol emitters in extending the shelf-life of three convenience-style food products were investigated. 

## 2. Experimental Section

### 2.1. Optical O_2_ Sensor and Analysis

Optical O_2_ sensors were prepared by using well known Platinum octaethylporphyrin-ketone (Pt-OEPK) (Luxcel Bioscience, Cork, Ireland), spotted (4 μL) on Durapore paper (Millipore Inc., Bedford, MA, USA), allowed to dry and cut to size of 5 mm. Sensors were then attached to stickers (Avery price marking stickers, California, CA, USA) for adhesion to the underside of packaging laminates. Sensors were read using a Mocon Op-Tech O_2_ Platinum (Mocon Inc., Minneapolis, MN, USA) measurement device, which allows for instant oxygen readings ranging from 0.001% to 25% O_2_ in 0.5 s. This system allows for the handheld instrument to be transportable with the use of a portable computer with Mocon Op-Tech software and complies with standards ASTM F2714. Instrumentation underwent calibration using a Cal-Card, where a simple gas-free method of calibration was carried out using two scan zones of 0% O_2_ and air. All food packs described in this study contained O_2_ sensors and all packaging samples were read daily using this non-destructive method.

### 2.2. Ethanol Emitter Preparation

Ethanol emitters (EE) were used as an in pack antimicrobial sachet. They were prepared in house, by using 3 mL of alcohol gel (Selden, Derbyshire, UK) and placed in pouches formed by using Excell LDPE polymer films (supplied by Fispak, Dublin, Ireland) and heat-sealed using a Henkelman Polar 80 vacuum packer and sealer (Henkelman BV, Hertogenbosch, NL, USA). The use of gel-based alcohol provided for the slow release of ethanol vapour into the packaging headspace. Pouches were micro perforated before being placed into food packs to allow for the release of ethanol over time in the headspace of packaging.

### 2.3. Sample Preparation

Pre-cooked convenience-style foods were made available from an Irish food manufacturer. Products, including; pre-cooked bacon slices (BA), battered sausages (BS) and beef and potato pies (PP) were selected to monitor the efficiency of the packaging process through the shelf-life evaluation of these products as the company in question had highlighted these products as being problematic in terms of reduced shelf-lives due to mycological growth. All samples were packed in thermoformed retail-ready 2 mm thick PS/EVOH/PE trays (250 mm × 170 mm) (<1 cm^3^/m^2^/24 h O^2^ permeability at 20 °C) and contained through the application of a high barrier lidding laminate Cryovac ULM491 (<1 cm^3^/m^2^/24 h O^2^ permeability at 4 °C) at 43 μm thickness which was heat-sealed to the tray after product filling. Each product varied in unit pack content. Table 1 highlights the number of product units present in each pack, for each commercial product-type. Repeat samples were also prepared with ethanol emitters (EE) placed in packs and compared. Packaging was carried out using an FP Speedy 2 (ILPRA, Italy) packaging station, sealing two trays per cycle. A combined process of vacuum (1–2 s) application followed by gas flushing and sealing (2.5 s) using a gas mix of 30% CO_2_ and 70% N_2_ (BOC gases, Ireland), process designed to exclude in-pack O_2_. The level of vacuum pressure and gas fill was carried out to manufacturers packaging settings listed in Table 1. Unit specifications showed that the equipment had a pressure capacity which ranged from 0 to 0.60 MPa (Max.) for vacuum and gas fill. Table 1 also presents the new packaging settings selected for the shelf-life optimisation study. Samples packaged under normal conditions were prepared with the use of a sensor which was pre-attached to laminate materials prior to entering the packaging process. All samples were monitored over time to determine the level of O_2_ present immediately following pack sealing and following its removal from the processing line. Subsequently, repeat samples were produced to compare the effects of applying a range of different pressure settings for both the vacuum and gas flushing processes on line and determining the impact of these process modifications on O_2_ levels in product packs. All samples were refrigerated at 4 °C immediately after packaging.

**Table 1:** Sample list with packaging settings (Vacuum—V/Gas—G) expressed in MPa.

Sample	Abbreviation	No. of Units	Original Packaging	New Packaging
		per Pack	Settings (MPa)	Settings (MPa)
Battered Sausages	BS	6	V (0.20)/G (0.06)	V (0.35)/G (0.02)
			or V (0.01)	
Bacon Slices	BA	8	V (0.01)	V (0.35)/G (0.02)
Beef & Potato Pie	PP	4	V (0.01)	V (0.35)/G (0.02)

### 2.4. Microbial Analysis

Microbial testing was carried on samples for Total Viable Counts (TVC) and yeasts and moulds. Samples were tested on a weekly basis for 5 weeks (35 days). TVC was determined using total viable count agar (Sigma-Aldrich, MO, USA), with dilutions of 10^1^–10^6^ and incubated at 30 °C for 48 h. Limits for total viable count (TVC) were log_10_ 6 [27]. Yeast and mould counts were assessed using dilutions described above and plated on compact dry yeast and mould plates (Hyserve, Uffling, Germany) and incubated at 25 °C for 7 days. Colonies were counted and presented in log_10_ cfu/g sample and limits were exceeded when mould counts reached 10^5^/g or 5 log10 (cfu/g) [27]. 

### 2.5. Sensory Analysis Design

Sensory analysis was carried out to determine if the use of EE had an effect on product quality perception. Twenty-six panellists were chosen form University College Cork, Ireland to partake in the study. The selection criteria for panellists included; availability to attend on each day of the study, motivation and were regular consumers of ready-cooked products, especially product types similar to those being assessed in this study. Panellists were all aged between 21 and 40 and consisted of a 50:50 male and female balance. Testing was carried out in accordance with ISO standards [28], where individual booths were provided and samples were assigned random three digit codes for blind assessment. Panellists were asked to rate descriptors on a ten point scale. A list of descriptors used for products can be seen in Table 2. Sensory analysis was assessed on day 14 and 35, to allow maximum exposure of foods to ethanol over a 35-day shelf-life. Panellists were provided with six samples, consisting of three products packaged at new packaging settings and replicates packaged with ethanol emitters. Samples were presented in a cooked state as instructed by pack guidelines.

### 2.6. Statistical Analysis

**Figure 1:**
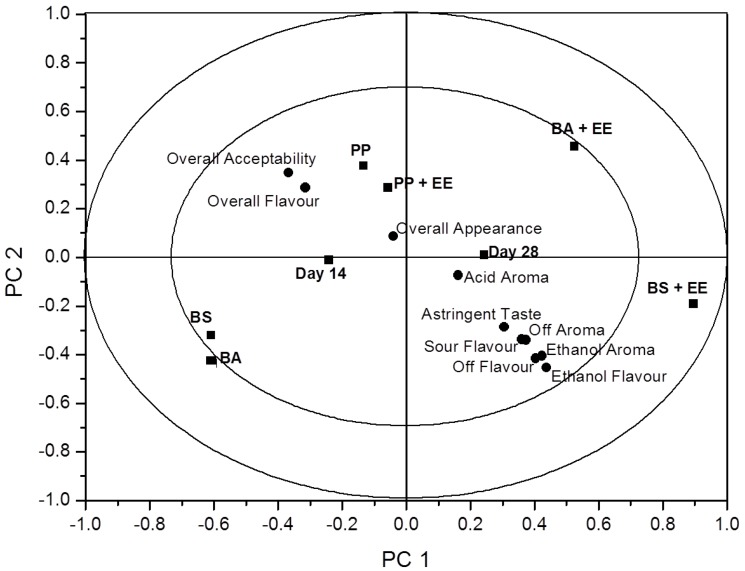
An overview of the variation found in the mean data from the ANOVA-partial least squares regression (APLSR) correlation loadings plot for each of the eight treatment groups assessed by a trained sensory group. Shown are the loadings of the *X*- and *Y*-variables for the first two principle components (PC’s) for averaged data validated over replicates. ■ = sample and days of analysis; ● = sensory descriptor.

Raw data was accumulated from sensory evaluation and processed using ANOVA-partial least squares regression (APLSR). The optimal number of components in the APLSR models presented was determined to be two principle components (Figure 1). Principle component (PC) 1 *versus* PC 2 is presented, as other PC’s did not yield any additional information. The validated explained variance for the model constructed was 18.52% and the calibrated variance was 25.43%. To derive the significance indictors for the relationships determined in the quantitative APLSR, regression coefficients were analysed by Jack-Knifing (Table 3). All analyses were performed using the Unscrambler Software, version 7.6 (Camo ASA, Trondheim, Norway).

**Table 2:** List of descriptors for sensory analysis.

Attribute	Description
Overall Appearance	0 = Extremely Dislike, 10 = Extremely Like
Off Aroma	0 = None, 10 = Extreme
Ethanol Aroma	0 = None, 10 = Extreme
Acid Aroma	0 = None, 10 = Extreme
Overall Flavour Liking	0 = Extremely Dislike, 10 = Extremely Like
Off Flavour	0 = None, 10 = Extreme
Sour Flavour	0 = None, 10 = Extreme
Astringent Taste	0 = None, 10 = Extreme
Ethanol Flavour	0 = None, 10 = Extreme
Overall acceptability	0 = Extremely Unacceptable, 10 = Extremely Unacceptable

**Table 3:** Significance of estimated regression coefficients (ANOVA *p*-values) for the relationships of sensory terms as derived by jack-knifing uncertainty testing of ready cooked foods.

	Sample						Time	
	BS	BS + EE	PP	PP + EE	BA	BA + EE	Day 14	Day 35
Overall Appearance	0.93 ns	−0.33 ns	0.27 ns	0.23 ns	0.47 ns	0.78 ns	0.64 ns	0.58 ns
Off Aroma	−0.003 **	0.001 ***	0.001 ***	−0.004 **	−0.85 ns	−0.001 ***	−0.40 ns	0.42 ns
Ethanol Aroma	0.001 ***	0.001 ***	0.001 ***	−0.003 **	−0.90 ns	−0.001 ***	−0.61 ns	0.64 ns
Acid Aroma	−0.04 *	0.02 *	−0.19 ns	−0.17 ns	−0.53 ns	−0.28 ns	−0.59 ns	0.63 ns
Overall Flavour Liking	0.002 **	0.001 ***	0.002 **	0.004 **	0.99 ns	0.001 ***	0.67 ns	−0.61 ns
Off Flavour	−0.002 **	0.001 ***	−0.003 **	−0.005 **	−0.74 ns	−0.001 ***	−0.56 ns	0.38 ns
Sour Flavour	−0.003 **	0.001 ***	−0.002 **	−0.007 **	−0.83 ns	−0.001 ***	−0.48 ns	0.46 ns
Astringent Taste	−0.002 **	0.001 ***	−0.015 *	−0.02 *	−0.94 ns	−0.001 ***	−0.81 ns	0.77 ns
Ethanol Flavour	0.001 ***	0.001 ***	0.001 ***	−0.002 **	−0.65 ns	−0.001 ***	−0.48 ns	0.37 ns
Overall Acceptablility	0.003 **	0.001 ***	0.001 ***	0.002 **	0.75 ns	0.001 ***	0.45 ns	−0.42 ns

ns = not significant; * *p* < 0.05; ** *p* < 0.01; *** *p* < 0.001.

## 3. Results and Discussion

### 3.1. Packaging Assessment and Optical O_2_ Readings

**Figure 2:**
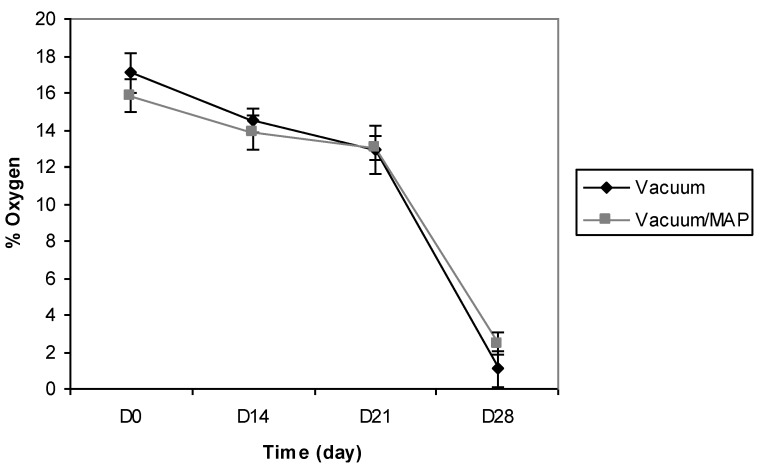
Mean O_2_ profiles for battered sausages (BS) packaged under vacuum or packaged under a combination of vacuum and modified atmosphere packaging (MAP); vacuum (0.01 MPa) and vacuum/MAP (0.20/0.06 MPa).

The research undertaken in this study was conducted, in conjunction with industrial involvement, to ascertain packaging performance for a range of convenience-style food products which were processed and packaged to meet a 28-day retail-required shelf-life. The company partner involved in this research employed two basic packaging approaches to pack all manufactured products; one which pulled a vacuum (0.01 MPa) around trayed-products prior to heat-sealing and, the second which pulled a vacuum (0.20 MPa), followed by gas flushing (0.06 MPa) prior to heat-sealing. These two packaging approaches were chosen for initial study to monitor O_2_ presence within packs. To this end, BS were chosen as a test product for assessment by both packaging approaches, *i.e.*, BS using vacuum tray packaging only (0.01 MPa) and, BS using vacuum (0.20 MPa) and gas flush tray packaging (0.06 MPa). Continuous non-destructive monitoring of O_2_ by optical sensors throughout product storage showed that both commercial packaging methods employed were quite poor in terms of removing O_2_ from food packs to achieve the commercially-desired and expected oxygen-less state. The mean O_2_ profiles of BS packaged using the two packaging approaches described are presented in Figure 2. The vacuum method (0.01 MPa) only reduced the mean O_2_ level within packs to 17.5% (just slightly lower than normalised levels found in atmospheric air—21%). This level of O_2_ in food packs is undesirable as it can lead to elevated microbial growth and oxidation reactions resulting in a product with a shorter than expected or required shelf-life [23,29]. During product storage, it was observed that O_2_ declined by 9% by day 24 and this coincided with the appearance of visual mould growth on BS. This resulted in a shorter shelf-life than required by the manufacturer. The combined use of vacuum and MAP produced similar results to that determined for the vacuum-only packaging treatment, with O_2_ levels in packs immediately post packaging determined as 16%. Again, the gradual decline in O_2_ for the vacuum and MAP combination over time closely matched that of the vacuum packaging only profile. Consequently, the overall finding from this preliminary product storage trial was that the commercial packaging approaches used to package BS products in no way came near to achieving targeted in-pack O_2_ levels [18].

Equipped with the determined knowledge that O_2_ levels in BS packs were much greater than expected or desired, a range of packaging treatments were chosen to ascertain if altering packaging regimen had any impact on the O_2_ levels remaining in packs post packaging. This was undertaken by altering the packaging pressures applied when using various vacuum/MAP combinations for use again with BS products. The mean O_2_ levels present in product packs under these varying packaging treatments, immediately after packaging and throughout product storage, are shown in Table 4.

**Table 4:** Mean O_2_ levels (%) found in BS packs held under varying packaging treatments (standard deviation ≤ 0.50%). V—represents the level of vacuum pressure applied (MPa); G—represents the level of gas flushing pressure applied (MPa).

							Differential
Sample	D0	D7	D14	D21	D28	D35	D0–D35
V (0.01)/G (0.02)	14.7	13.5	13.3	12.5	10.4	4.1	10.7
V (0.04)/G (0.02)	15.4	13.7	13.5	13.5	12.6	5.0	10.4
V (0.08)/G (0.02)	14.7	13.5	13.2	12.4	11.8	7.0	7.7
V (0.20)/G (0.02)	12.2	11.5	10.4	9.8	8.1	6.8	5.4
V (0.35)/G (0.02)	8.2	6.9	6.7	6.3	6.0	4.5	3.7
V (0.60)/G (0.02)	8.1	6.5	6.3	6.1	6.0	4.4	3.7
V (0.01)/G (0.03)	14.3	13.5	13.1	12.2	10.1	4.0	10.3
V (0.04)/G (0.03)	15.1	13.7	13.5	13.5	12.6	5.0	10.1
V (0.08)/G (0.03)	15.0	13.5	13.0	12.2	11.5	7.1	7.9
V (0.20)/G (0.03)	11.4	11.4	10.1	9.5	7.8	6.8	4.6
V (0.35)/G (0.03)	8.4	6.7	6.5	6.3	6.1	4.6	3.8
V (0.60)/G (0.03)	8.2	6.4	6.3	6.0	5.8	4.5	3.7

The combination of vacuum/MAP levels and their effect on O_2_ levels were shown to vary greatly over the 35 day storage trial. In general, the application of high vacuum levels lowered initial O_2_ concentrations in packs on day 0. Vacuum pressures applied at 0.35 and 0.60 MPa removed the greatest volume of O_2_ from packs, averaging 8.3% O_2_ post-packaging. The increase in MAP fill pressure from 0.02 to 0.03 MPa appears to have had a negligible effect on O_2_ levels. From this study, it was determined that the application of the packaging treatment V (0.35)/G (0.02) was optimal for the packaging of rigid tray formats of BS. Additionally, the reduced O_2_ levels in packs also eliminated the presence of visual mould on products up to 35 days of product storage. Therefore, it was concluded that this packaging treatment should be applied to a number of food products in order to see whether or not the packaging modifications applied could lower O_2_ levels in other product packs and more importantly, extend the shelf-life of these products to greater than 28 days. The use of a lower gas flush setting (0.20) was selected for further studies as it appeared that no further improvement in O2 levels was achieved by increasing this level further, thereby saving on gas utilization and cycle time per tray. The 0.35 MPa vacuum treatment was also chosen for further studies as it requires less time per packaging cycle and produced equivalent results when compared against higher vacuum pressures.

### 3.2. Revised Packaging Treatment

A total of three convenience-style ready-cooked food products were chosen for shelf-life studies to ascertain if the new packaging treatment (V (0.35)/G (0.02)) was suitable for maintaining an acceptable product with a shelf-life of 28 days. As previously described retail products consisting of battered sausages (BS), beef and potato pie (PP) and bacon slices (BA) were chosen for assessment. All samples were prepared as described previously, using an optical O_2_ sensor to monitor O_2_ levels throughout storage. The new packaging treatment (V (0.35)/G (0.02)) was also compared against the original commercial packaging treatments used at the start of these studies (vacuum application (0.01 MPa) and vacuum application (0.20 MPa), followed by gas flushing (0.06 MPa). The use of ethanol emitters (EE) was added to duplicate treatments of the above to determine if further shelf-life extension could be achieved for all products (beyond 28 days) using these active packaging components. The mean levels of O_2_ recorded for all experimental treatments over time are shown in Table 5. Packaging treatment V (0.35)/G (0.02) had reduced levels of O_2_ immediately post packaging and throughout storage when compared to the original commercially-used packaging procedures. In the case of BS the O_2_ differential was reduced from 15.7% to 2.5% O_2_ using the new settings. This trend continued for the other product types examined, where the initial concentration of in-pack O_2_ was lowered following the application of the newly modified packaging settings. Product packs containing EE provided an antimicrobial effect which was signalled by a lack of O_2_ utilisation in packs over time.

### 3.3. Microbial Analysis

Microbial analysis was carried out on a weekly basis to establish the mould counts present in all sample treatments. It was identified that mould was the primary microbial spoilage mechanism across the product range. Table 6 presents the log10 mould counts from days 0 to 35. On days 0, 7 and 14, near identical counts were observed across all product treatments, yielding no trends or significant differences between treatments. Day 21 shows the first notable difference across all treatments. It is clear that all samples are still within acceptable limits at day 21. The normal commercial packaging format employed at the start of this study for each of the three products examined exceeded yeast/mould limits by day 28. However, the application of the new packaging settings was seen to extend shelf-life for all products up to day 35 of storage. It can be noted that the use of EE in all samples further extended shelf-life by an additional 7 days (day 42) before exceeding limits, compared to samples packed under the new packaging conditions (V (0.35)/G (0.02)). The ability to extend shelf-life further could benefit product manufacturers, distributors and retailers who require longer shelf-lives, as transportation distances to market increase and waste minimization measures become more demanding.

**Table 5:** Mean O_2_ levels found in all samples over shelf life study (including standard deviation). V—represents the level of vacuum pressure (MPa); G—represents the level of gas flush (MPa).

Sample	Time (Day)						Differential %
	D0	D7	D14	D21	D28	D35	D0–D35
BS Normal 1 (V (0.01))	16.5 ± 0.81	13.2 ± 0.74	13.8 ± 0.75	12.6 ± 0.57	8.6 ± 0.62	0.9 ± 0.44	15.7
BS Normal 1 (V (0.01)) + EE	16.6 ± 0.70	15.3 ± 0.83	14.6 ± 0.52	13.9 ± 0.66	10.4 ± 0.87	5.7 ± 1.20	10.9
BS Normal 2 (V (0.20)/G (0.06))	12.9 ± 1.00	11.1 ± 0.91	10.2 ± 0.61	9.7 ± 0.83	6.4 ± 0.76	4.1 ± 0.86	8.8
BS Normal 2 (V (0.20)/G (0.06)) + EE	12.8 ± 0.90	11.3 ± 0.40	10.4 ± 0.64	9.9 ± 0.85	8.7 ± 0.57	6.1 ± 0.77	6.7
BS (V (0.35)/G (0.02))	7.5 ± 0.39	6.7 ± 0.48	6.8 ± 0.32	6.8 ± 0.36	7.0 ± 0.20	5.0 ± 0.27	2.5
BS (V (0.35)/G (0.02)) + EE	8.4 ± 0.41	6.8 ± 0.37	6.7 ± 0.38	6.5 ± 0.44	6.7 ± 0.22	7.4 ± 0.11	1.0
PP Normal (V (0.01))	14.1 ± 1.10	13.1 ± 0.83	12.5 ± 0.64	8.4 ± 0.76	2.7 ± 0.46	0.9 ± 0.62	13.2
PP Normal (V (0.01)) + EE	14.4 ± 0.94	13.8 ± 0.73	12.9 ± 0.88	9.4 ± 0.72	6.4 ± 0.65	4.7 ± 0.80	10.7
PP (V (0.35)/G (0.02))	7.3 ± 0.51	6.5 ± 0.47	6.4 ± 0.47	6.3 ± 0.56	5.5 ± 0.32	2.2 ± 0.43	5.1
PP (V (0.35)/G (0.02)) + EE	6.9 ± 0.33	6.6 ± 0.38	6.8 ± 0.13	5.9 ± 0.23	6.0 ± 0.27	4.3 ± 0.11	2.7
BA Normal (0.01)	16.9 ± 1.31	16.5 ± 0.73	16.4 ± 0.33	15.3 ± 0.37	14.4 ± 0.64	8.2 ± 0.89	10.7
BA Normal (0.01) + EE	16.8 ± 1.00	16.5 ± 0.84	16.2 ± 0.76	15.8 ± 0.53	15.1 ± 0.45	11.7 ± 0.44	5.1
BA (V (0.35)/G (0.02))	9.5 ± 0.45	7.9 ± 0.53	7.7 ± 0.45	7.7 ± 0.27	7.7 ± 0.36	7.9 ± 0.40	1.6
BA (V (0.35)/G (0.02)) + EE	7.1 ± 0.22	6.3 ± 0.46	6.1 ± 0.32	6.1 ± 0.13	6.8 ± 0.12	6.3 ± 0.18	0.9

The utilisation of EE showed great potential in extending product shelf-life without the need for more advanced processing or packaging equipment or materials. It has been observed that EE have the ability to dramatically extend shelf-life in food products [30]. Total viable count (TVC) limits of log_10_ 6 [27] were not reached in any product sample packaged using the new experimental settings, either with or without EE by day 35.

**Table 6:** Mean log10 mould counts for varying treatments (MPa) across three products, n/d—not determined due to ^exceeding limits (10^5^/g or 5 log10 (cfu/g) [31]) or presence of visual mould.

Sample	Time						
	D0	D7	D14	D21	D28	D35	D42
BS Normal 1 (V (0.01))	2.8 ± 0.14	3.2 ± 0.21	3.9 ± 0.32	4.5 ± 0.17	^6.1 ± 0.10	n/d	n/d
BS Normal 1 (V (0.01)) + EE	2.9 ± 0.09	3.1 ± 0.15	3.7 ± 0.35	4.0 ± 0.22	^5.1 ± 0.09	n/d	n/d
BS Normal 2 (V (0.2)/G (0.06))	2.6 ± 0.25	3.0 ± 0.07	3.5 ± 0.23	4.3 ± 0.27	^5.8 ± 0.10	n/d	n/d
BS Normal 2 (V (0.2)/G (0.06)) + EE	2.8 ± 0.18	3.2 ± 0.21	3.4 ± 0.32	3.8 ± 0.19	^5.0 ± 0.28	n/d	n/d
BS (V (0.35)/G (0.02))	1.8 ± 0.29	2.2 ± 0.45	2.7 ± 0.49	2.9 ± 0.34	3.6 ± 0.37	4.8 ± 0.07	^6.0 ± 0.29
BS (V (0.35)/G (0.02)) + EE	1.7 ± 0.27	1.8 ± 0.29	2.0 ± 0.49	2.3 ± 0.27	3.1 ± 0.32	4.1 ± 0.29	4.8 ± 0.12
CB Normal (V (0.01))	2.8 ± 0.20	3.2 ± 0.26	3.7 ± 0.15	4.2 ± 0.18	^6.8 ± 0.11	n/d	n/d
CB Normal (V (0.01)) + EE	2.7 ± 0.28	3.2 ± 0.37	3.6 ± 0.41	3.9 ± 0.50	^5.2 ± 0.07	n/d	n/d
CB (V (0.35)/G (0.02))	1.7 ± 0.17	2.1 ± 0.12	2.4 ± 0.07	2.8 ± 0.04	3.4 ± 0.16	4.5 ± 0.21	^5.4 ± 0.29
CB (V (0.35)/G (0.02)) + EE	1.5 ± 0.04	2.0 ± 0.13	2.2 ± 0.09	2.5 ± 0.11	3.0 ± 0.27	4.4 ± 0.31	^5.0 ± 0.42
BA Normal (V (0.01))	2.9 ± 0.10	3.1 ± 0.16	3.6 ± 0.19	4.2 ± 0.14	^6.8 ± 0.08	n/d	n/d
BA Normal (V (0.01)) + EE	2.8 ± 0.06	3.0 ± 0.12	3.2 ± 0.22	3.8 ± 0.15	^5.6 ± 0.12	n/d	n/d
BA (V (0.35)/G (0.02))	1.4 ± 0.07	2.0 ± 0.09	2.4 ± 0.17	2.9 ± 0.21	3.7 ± 0.04	4.8 ± 0.05	^5.3 ± 0.17
BA (V (0.35)/G (0.02)) + EE	1.2 ± 0.05	1.3 ± 0.08	1.6 ± 0.14	1.9 ± 0.18	2.4 ± 0.10	3.9 ± 0.07	4.8 ± 0.11

### 3.4. Sensory and Statistical Analyses

EE were used in selected product packs in order to ascertain if they could potentially extend shelf-life. The continued presence of ethanol in the headspace of the packs was assessed to see if product taste or aroma was affected during sensory assessment of these products. A total of 26 panellists were provided with 6 samples, three products that were packaged both with and without EE. Questionnaires designed with a range of descriptors were rated by panellists to best describe the taste and aroma profile of each sample. Table 3 represents the significance of sensory relationship terms; the sign indicates whether significance is positively or negatively correlated. Findings from this study show that only one of the six products assessed were found to be unacceptable. BS + EE were found to impart negative effects on flavour and aroma, where ethanol aroma and flavour were negatively and significantly correlated (*p* < 0.001). Other samples continued to be acceptable to panellists where PP + EE and BA + EE were found to have an overall significant (*p* < 0.01) liking for flavour and overall acceptability (*p* < 0.001). This would lead to the belief that the continued presence of ethanol in samples had no effect in two of the three EE-containing samples. The negative results associated to BS + EE could be due to the increased level of fat associated with this particular product, causing an off flavour (*p* < 0.001) and imparting an ethanol aroma (*p* < 0.001). In the case of BS the sample prepared without EE was found to be significantly acceptable compared to sample BS + EE. The use of EE in food products has been seen to have no effect on taste and/or aroma in bread products [29,30]. Figure 1 represents an overview plot of the mean data from the ANOVA correlation values for all six samples. Principal component 1 *versus* principal component 2 shows the arrangement of descriptors and samples. The presence of negative descriptors (ethanol aroma and flavour, sour flavour and astringent taste can be seen to be strongly correlated with the sample BS + EE, where we see the only noted unacceptable sample affected by ethanol in ANOVA *p*-values. The ability to extend shelf-life using EE without reducing the sensory quality of certain food products shows great potential of active packaging technologies. These technologies can allow foods to be stable for long periods of time without the need for further processing and the addition of food preservatives and additives during manufacture.

Results showed that the use of EE in a variety of ready-cooked, convenience-style foods have a positive effect in extending shelf-life without being accompanied by a decline in perceived product quality. The use of such antimicrobial sachets could provide industry to evaluate what packaging techniques are adopted. The use of EE is without a doubt one of the most exciting interactive packaging technologies available to the food industry [32], however, the technology is grossly underutilised in commercial retail packs of food today.

## 4. Conclusions

Optical O_2_ sensors were shown to readily integrate with commercial packaging of ready-cooked, convenience-style food products. The ability to non-destructively measure O_2_ immediately after packaging and throughout shelf life was reported. O_2_ levels in excess of that intended was clearly recorded. Vacuum and MA packaging methods were shown to have O_2_ levels in excess of 15%. Alteration of packaging settings were monitored for changes in these O_2_ levels and resulted in a decrease of O_2_ levels to 8%. Best performance settings were selected by results obtained by O_2_ sensor readings resulting in a new packaging setting for improved product containment. Although O_2_ is clearly available for degradative processes within packs, the lowered O_2_ levels obtained maintained an acceptable product for longer than the required shelf-life. The use of EE further extended product shelf-life through antimicrobial control without adversely affecting overall acceptance of product quality.
